# *Notes from the Field:* The First 100 Days of Five Ebola Outbreaks — Democratic Republic of the Congo, Uganda, and West Africa, 2007–2026

**DOI:** 10.15585/mmwr.mm7537e1

**Published:** 2026-09-24

**Authors:** John Rossow, Thomas C. McHale, James Fuller, Delayo L. Zomahoun, Amy Boore, Lauren Erickson-Mamane, Hailey Whitmire, Jaymin C. Patel, Dumazedier K. Kabasele, Noemi B. Hall, Issaka Kabore, Billy Mutombo Mpianga, Katrin S. Sadigh, Amy Schuh, Tara Sealy, Erika Meyer, Elissa Meites, Christine Atherstone, Benjamin A. Dahl, CDC 2026 Ebola Response 100-Day Reporting Team, CDC 2026 Ebola Response 100-Day Reporting Team, Andrew T. Boyd, Anna Bratcher, Vance Brown, Sean Browning, Jonathan Bryant-Genevier, Mary J. Choi, Athalia Christie, Matthew Cole, Amber Dismer, Carrie Eggers, Sascha Ellington, Maestro Evans, Neil Gupta, Ruth Kallay, John Klena, Timothee Kinkela, Michael H. Kinzer, Veronica Lea, Emmanuel A. Mensah, Mpingulu Minlangu, Joel M. Montgomery, Michael Park, Nicole Paterson, Natalie Peters, Satish K. Pillai, Anne Purfield, Logan Ray, Jessica N. Ricaldi, Philip Ricks, Daniel Rosen, Dean Sayre, Rachel B. Slayton, Trevor Shoemaker, Ciara Sugarman, Leisel E. Talley, Alison M. Todres, Henry Walke, Emily Weston, Kristina Wielgosz, Emily Zielinski-Gutierrez

**Affiliations:** ^1^CDC 2026 Ebola Response; ^2^U.S. Public Health Service, Rockville, Maryland.; CDC; CDC; CDC; CDC; CDC; CDC; CDC; CDC; CDC; CDC; CDC; CDC; CDC; CDC; CDC; CDC; CDC; CDC; CDC; CDC; CDC; CDC; CDC; CDC; CDC; CDC; CDC; CDC; CDC; CDC; CDC; CDC; CDC; CDC; CDC; CDC; CDC; CDC; CDC; CDC.

**Summary**

**What is already known about this topic?**

The Democratic Republic of the Congo (DRC) is experiencing its largest and deadliest Ebola disease outbreak, which is also the largest Ebola outbreak caused by Bundibugyo virus and the second largest Ebola outbreak worldwide.

**What is added by this report?**

The 2026 Ebola outbreak resulted in 5,458 confirmed cases and 2,606 deaths in DRC in the first 100 days after initial detection, indicating rapid growth. No previous Ebola outbreak caused >800 cases during the first 100 days.

**What are the implications for public health practice?**

This Ebola outbreak appears to be expanding faster than any previously documented Ebola outbreak, with seven times as many cases 100 days after initial detection than the 2014 Ebola outbreak in West Africa, the largest outbreak worldwide. Urgent implementation of public health interventions to identify cases, trace contacts, and limit transmission are needed to bring the outbreak under control.

The first 100 days after identification of an outbreak are important to understanding transmission dynamics, impact of early public health interventions, and trajectory of potential future cases and deaths. A large Ebola disease outbreak in the Democratic Republic of the Congo (DRC), which has become the country’s largest and deadliest, is ongoing. To better understand the current outbreak, this report compared metrics from the first 100 days of this outbreak with those of four past Ebola outbreaks. The historic outbreaks of Ebola disease selected for this comparison include the two largest (the 2014 outbreak in West Africa and a 2018 outbreak in DRC), and the only two previous outbreaks caused by Bundibugyo virus (the 2007 outbreak in Uganda and the 2012 outbreak in DRC). This activity was reviewed by CDC, deemed not research, and conducted consistent with applicable federal law and CDC policy.*

## Investigation and Outcomes 

### Epidemiology of Five Outbreaks

**2007 Bundibugyo virus disease outbreak, Uganda.** Bundibugyo virus (species *Orthoebolavirus bundibugyoense*) was first identified in 2007 when it caused an outbreak of Ebola disease in Uganda (*1*). Fewer than 50 cases were identified during the first 100 days. This outbreak was limited to one district in Uganda (Bundibugyo district) and ended within 6 months, with a total of 131 confirmed, probable, and suspected cases and 42 (32%) deaths in persons with confirmed, probable, or suspected Ebola disease.

**2012 Bundibugyo virus disease outbreak, DRC.** The second known Bundibugyo virus outbreak (species *O. bundibugyoense*) occurred in 2012 in DRC; fewer than 50 cases were identified during the first 100 days. This outbreak was limited to a single health zone in DRC (Isiro health zone, Haut-Uélé province) and ended within 6 months, with 62 confirmed, probable, and suspected cases and 34 (55%) deaths in persons with confirmed, probable, and suspected Ebola disease.

**2014 Ebola virus disease outbreak, West Africa.** In 2014, a large outbreak of Ebola virus disease caused by Ebola virus (species *Orthoebolavirus zairense*) was declared in Guinea. The initial outbreak report included 49 confirmed cases and 29 (59%) deaths. During the first 100 days (March 23–July 1, 2014), 759 suspected, probable, or confirmed cases and 467 (62%) deaths in persons with suspected, probable, or confirmed Ebola virus disease were reported from Guinea, Liberia, and Sierra Leone. This outbreak lasted 3 years, resulting in 28,610 cases and 11,308 (40%) deaths across 10 countries, becoming the largest Ebola disease outbreak in history (Figure) (*2*).

**FIGURE:**
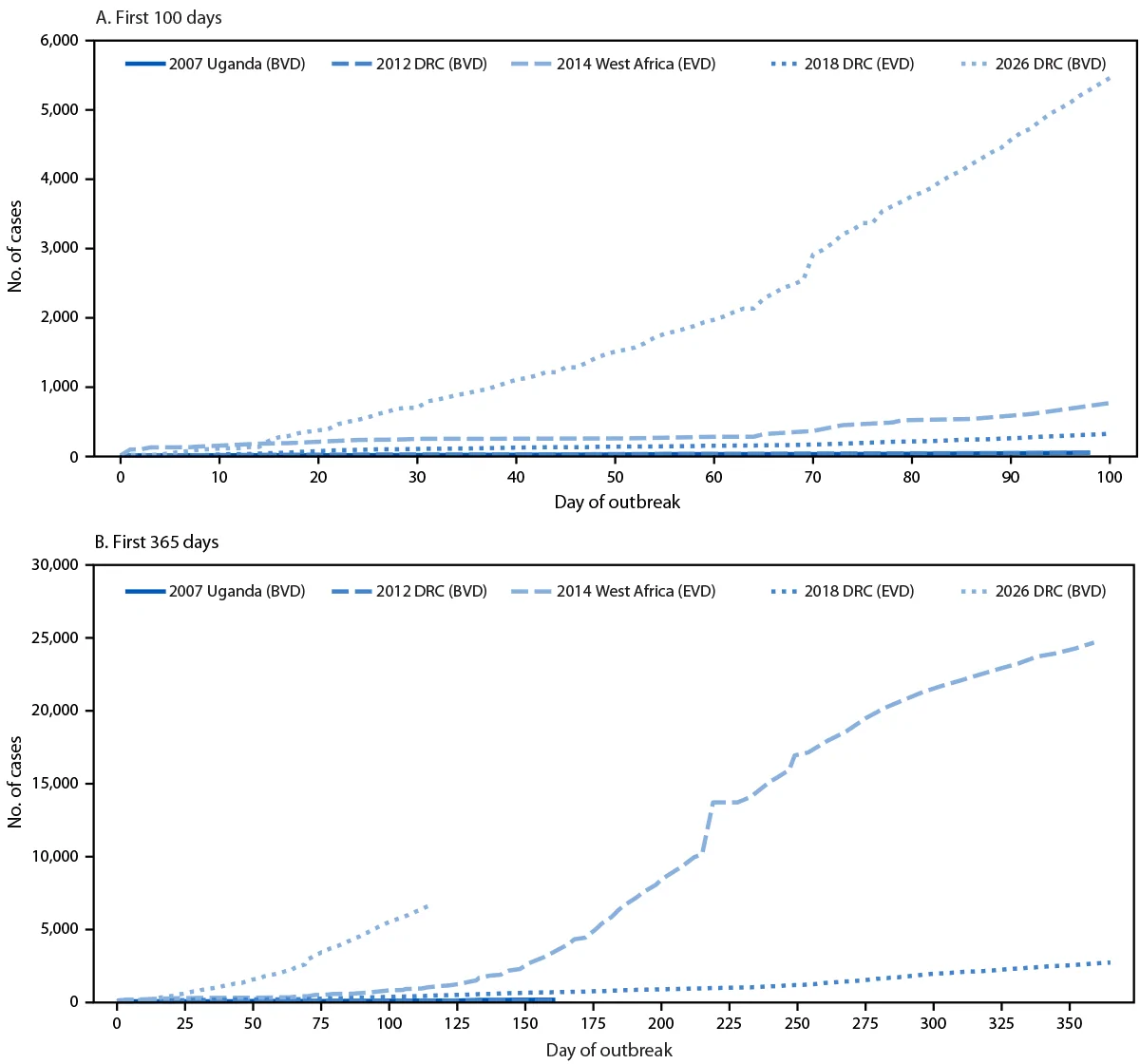
Cumulative number of Ebola disease cases,* by day, for the first 100 days (A) and the first year (B) of the outbreak — five Ebola outbreaks, Democratic Republic of the Congo, Uganda, and West Africa, 2007–2026 **Abbreviations**: BVD = Bundibugyo virus disease; DRC = Democratic Republic of the Congo; EVD = Ebola virus disease. * The 2026 outbreak includes confirmed cases, the 2018 outbreak includes confirmed and probable cases, and the other outbreaks include confirmed, probable, and suspected cases of Ebola disease.

**2018 Ebola virus disease outbreak, DRC.** In 2018, a large outbreak of Ebola virus disease caused by Ebola virus (species *O. zairense*) occurred in DRC. During the first 103 days (August 1–November 11, 2018), 295 confirmed cases, 38 probable cases, and 171 (51%) deaths among persons with confirmed Ebola disease were reported across 14 health zones, primarily in eastern DRC. This outbreak resulted in 3,470 cases and 2,287 (66%) deaths over a period of 22 months (*3*).

**2026 Bundibugyo virus disease outbreak, DRC.** On May 14, 2026, an outbreak of Ebola disease caused by Bundibugyo virus (species *O. bundibugyoense*) was detected in DRC. The outbreak was declared on May 15, at which time DRC’s Ministry of Health had identified 246 suspected cases and 76 suspected deaths, in addition to eight laboratory-confirmed cases and four deaths (*4*). During the outbreak’s first 100 days (May 14–August 21, 2026), 5,458 confirmed cases and 2,606 (48%) deaths were reported across 57 of DRC’s 519 health zones. Twenty-one probable or confirmed cases related to this outbreak were identified in Uganda, with limited local transmission, primarily among health care workers. One additional case, related to travel from DRC, was detected in France. During the first 100 days after detection, the current outbreak resulted in more cases than the other two largest Ebola disease outbreaks (7 and 16 times as many, respectively) (Figure). As of September 10, there has been no evidence of community transmission outside of DRC.

## Implications and Next Steps

During the first 100 days after its detection, the 2026 Ebola outbreak has grown faster than any previous Ebola disease outbreak. The current outbreak also had more cases at the time of detection than did other outbreaks. Detection of large numbers of cases at the time an outbreak is first declared can indicate that multiple chains of transmission are occurring and that virus was circulating widely before it was recognized. Although many variables could affect its future trajectory, considering different Ebola outbreaks’ first 100 days after detection and subsequent case numbers, the 2026 outbreak appears to be demonstrating rapid growth that arouses concerns about ongoing spread. 

The 2026 Ebola outbreak is proving to be challenging to control. This outbreak is occurring in areas affected by a protracted complex humanitarian emergency, including ongoing conflict involving armed groups, limited health care and other basic infrastructure, attacks on health workers, and community mistrust. These factors complicate the delivery of aid to affected communities despite substantial mobilization of response personnel and materials (e.g., laboratory equipment and personal protective equipment). 

The current outbreak trajectory underscores the urgent need to continue to scale up evidence-based Ebola control measures, such as rapid case and contact identification, expansion of diagnostic capacity, improvement of infection prevention and control in health care settings, adoption of safe and dignified burials, strengthening of cross-border surveillance and coordination, and community engagement (*2*). Analysis of administrative health zone–level Ebola response indicators (*5*) might help identify operational gaps and guide targeted interventions.

## Footnotes

* 45 C.F.R. part 46, 21 C.F.R. part 56; 42 U.S.C. Sect. 241(d); 5 U.S.C. Sect. 552a; 44 U.S.C. Sect. 3501 et seq.
